# Insights into the role of intracellular calcium signaling in the neurobiology of neurodevelopmental disorders

**DOI:** 10.3389/fnins.2023.1093099

**Published:** 2023-02-15

**Authors:** Benjamin Klocke, Kylie Krone, Jason Tornes, Carter Moore, Hayden Ott, Pothitos M. Pitychoutis

**Affiliations:** Department of Biology, University of Dayton, Dayton, OH, United States

**Keywords:** autism, SERCA2, ryanodine receptors, calcium, schizophrenia, attention-deficit hyperactivity disorder (ADHD), inositol triphosphate receptor (IP3)

## Abstract

Calcium (Ca^2+^) comprises a critical ionic second messenger in the central nervous system that is under the control of a wide array of regulatory mechanisms, including organellar Ca^2+^ stores, membrane channels and pumps, and intracellular Ca^2+^-binding proteins. Not surprisingly, disturbances in Ca^2+^ homeostasis have been linked to neurodegenerative disorders, such as Alzheimer’s and Parkinson’s diseases. However, aberrations in Ca^2+^ homeostasis have also been implicated in neuropsychiatric disorders with a strong neurodevelopmental component including autism spectrum disorder (ASD) attention-deficit hyperactivity disorder (ADHD) and schizophrenia (SCZ). While plasma membrane Ca^2+^ channels and synaptic Ca^2+^-binding proteins have been extensively studied, increasing evidence suggests a prominent role for intracellular Ca^2+^ stores, such as the endoplasmic reticulum (ER), in aberrant neurodevelopment. In the context of the current mini-review, we discuss recent findings implicating critical intracellular Ca^2+^-handling regulators such as the sarco-ER Ca^2+^ ATPase 2 (SERCA2), ryanodine receptors (RyRs), inositol triphosphate receptors (IP_3_Rs), and parvalbumin (PVALB), in the emergence of ASD, SCZ, and ADHD.

## 1. Introduction

Neurodevelopmental disorders (e.g., autism spectrum disorder; ASD, and attention-deficit hyperactivity disorder; ADHD) and schizophrenia (SCZ), a neuropsychiatric disorder with a strong neurodevelopmental component (Birnbaum and Weinberger, 2017; Seidman and Mirsky, 2017; Rund, 2018), comprise debilitating diseases that are highly variable in their symptomatology and etiology (McGrath et al., 2008; Christensen et al., 2016; Hansen et al., 2018; Sayal et al., 2018). These disorders arise due to the complex interplay between genetic risk factors and early life environmental stressors, including prenatal complications, malnutrition, hormone imbalance, and exposure to environmental toxins (e.g., neurotoxic metals) (Wetmore and Garner, 2010; Lord et al., 2018; Li et al., 2019; Ijomone et al., 2020). Recent research efforts have sought to identify common disrupted molecular mechanisms that may lead to abnormal neurodevelopment. One such candidate which has garnered interest is the disruption of intracellular calcium (Ca^2+^) homeostasis.

Intracellular Ca^2+^ concentration is critical for orchestrating numerous cellular processes, including signal transduction and gene expression (Bootman et al., 2001; Naranjo and Mellström, 2012; Bononi et al., 2013; Brini et al., 2014; Britzolaki et al., 2018). Consequently, Ca^2+^ mishandling is implicated in the pathophysiology of neurodegenerative disorders (e.g., Alzheimer’s and Parkinson’s diseases) (Pchitskaya et al., 2018), while recent evidence suggests that aberrations in intracellular Ca^2+^ signaling may also underlie abnormal neurodevelopment (Pourtavakoli and Ghafouri-Fard, 2022). Of the major neuronal Ca^2+^-handling players, plasma membrane voltage-gated Ca^2+^ channels (e.g., *Cacna1*) are well-reviewed with regards to their role in neurodevelopment (Breitenkamp et al., 2015; Cupertino et al., 2016; Pourtavakoli and Ghafouri-Fard, 2022). Readers are referred to recent excellent reviews discussing the implication of critical plasma membrane Ca^2+^ players (e.g., *CACNA1*) and Ca^2+^-binding proteins involved in synaptic release (e.g., Synaptotagmin) in the pathophysiology of brain disorders (Breitenkamp et al., 2015; Cupertino et al., 2016; Pourtavakoli and Ghafouri-Fard, 2022). Interestingly, dysfunction of endoplasmic reticulum (ER) Ca^2+^ regulators such as the sarco-ER Ca^2+^ ATPase 2 (SERCA2), which sequesters cytosolic Ca^2+^ into the ER, and the Ca^2+^-releasing channels inositol triphosphate receptors (IP_3_Rs) and ryanodine receptors (RyRs) have recently garnered interest in the pathophysiology of brain disorders (Britzolaki et al., 2018, 2020). In the context of the current mini-review, we discuss recent findings implicating aberrant ER-dependent Ca^2+^ homeostasis as a convergent pathophysiological mechanism in brain disorders with a strong neurodevelopmental component.

## 2. Autism spectrum disorders (ASD)

### 2.1. Ryanodine receptors (RyRs) and the fragile X messenger ribonucleoprotein 1 (FMR1)

**FIGURE 1:**
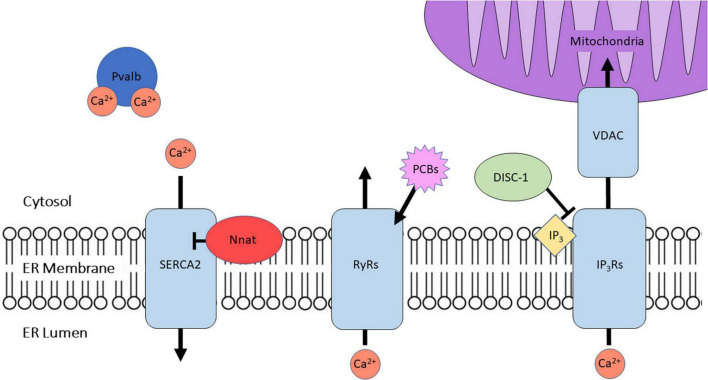
A summary of the proteins discussed herein and their primary role in regulating intracellular Ca^2+^ homeostasis. Proteins mediating ER Ca^2+^ efflux include ryanodine receptors (RyRs) and inositol triphosphate receptors (IP_3_Rs), while cytosolic Ca^2+^ is handled by the sarco-endoplasmic reticulum Ca^2+^ ATPase 2 (SERCA2), which is negatively regulated by neuronatin (Nnat). Parvalbumin (Pvalb) regulates cytosolic Ca^2+^ buffering *via* directly binding Ca^2+^ ions. Polychlorinated biphenyls (PCBs) target RyRs to exert their effects on Ca^2+^ homeostasis. IP_3_Rs associate with mitochondrial voltage-dependent anion channels (VDAC) in the mitochondrial-associated membranes (MAMs), and are regulated in part by disrupted in schizophrenia 1 protein (DISC-1).

Autism spectrum disorders is a neurodevelopmental disorder which comprises a wide array of behavioral symptoms including impaired sociability and communication skills, repetitive behaviors, and intellectual disability (Christensen et al., 2016; Lord et al., 2018). Although no single genetic factor is responsible for ASD, RyRs have been identified as a potential contributor to ASD pathology. RyRs are homotetrameric Ca^2+^-releasing channels expressed on the neuronal ER membrane; upon opening, the RyRs allow for the flux of Ca^2+^ ions from the ER stores into the cytosol (Figure 1; Abu-Omar et al., 2018). Notably, clinical studies suggest that mutations in genes coding for the different RyRs isoforms could possibly contribute to the pathophysiology of ASD. A copy number variation study has revealed a likely pathogenic duplication at 1q43, which encompasses the *RYR2* gene, thus identifying *RYR2* as a potential ASD risk gene (Soueid et al., 2016; Keil et al., 2019). Despite the fact that *Ryr3* has been shown to contribute to synaptic plasticity and cognitive flexibility in mice (Balschun et al., 1999), an earlier clinical study did not report an association between *RYR3* and ASD in a Japanese patient cohort (Tochigi et al., 2008). However, a more recent targeted sequencing and integrative analysis study of 3,195 Chinese patients with neurodevelopmental disorders exposed *RYR3* as one of the six novel candidate genes to preferentially contribute to ASD (Wang T. et al., 2021).

Preclinical studies have provided intriguing mechanistic insights into how RyR dysfunction could affect intracellular Ca^2+^ homeostasis and ASD-relevant phenotypes and endophenotypes in animal models. Interestingly, mutations in the *RYR1* and the fragile X messenger ribonucleoprotein 1 (*FMR1)* genes have both been associated with impaired Ca^2+^ signaling. Specifically, preclinical evidence suggests that the human T4826I*-RYR1* gain-of-function mutation and the human CGG-repeat expansion in the *FMR1* gene (i.e*., FMR1* premutation), are both associated with elevated intracellular Ca^2+^ signaling; indeed, the T4826I*-RYR1* gain-of-function mutation has been shown to result in increased intracellular Ca^2+^ concentrations in muscle cells (Barrientos et al., 2012), while murine cortical astrocytes with the *FMR1* premutation displayed enhanced asynchronous Ca^2+^ oscillations (Chen et al., 2010; Cao et al., 2013; Robin et al., 2017). Notably, Ca^2+^ signaling is critical for ensuring proper dendritic morphology and synaptic connectivity. Keil et al. (2019) assessed ASD-relevant behavioral and neurobiological correlates (i.e., dendritic morphology and social behavior) in adolescent mice with the humanized T4826I*-RYR1* gain-of-function mutation and with the *FMR1* premutation, as well as in double mutant (DM) mice (Keil et al., 2019). Interestingly, social deficits in T4826I male and DM female mice were both accompanied by abnormal dendritic morphology (Keil et al., 2019). Based on the authors, the observed changes in dendritic morphology in these mice could be attributed to altered intracellular Ca^2+^ dynamics, even though additional studies are needed to yield more conclusive results (Keil et al., 2019).

Sethi et al. (2021) conducted a follow-up study to understand the interaction of *Ryr1* and *Fmr1* and polychlorinated-biphenyls (PCBs) exposure in ASD-like behaviors (Sethi et al., 2021). PCBs comprise environmental contaminants with established neurodevelopmental consequences that exert their neurotoxic effects by binding to the RyRs (Pessah et al., 2010). In that study, dams were orally administered a PCB mixture from 2-weeks prior to mating until pup weaning (P21). Ultrasonic vocalizations at P7 were diminished in all three mutant pup genotypes while both male and female T4826I and DM pups exhibited high spontaneous grooming behavior (Sethi et al., 2021). Further, studies from the same group found that PCBs promote synaptogenesis in cultured hippocampal neurons, as evidenced by increased dendritic spines and miniature excitatory postsynaptic currents (Lesiak et al., 2014). Importantly, these effects were found to be RyR-dependent, as treatment with either the RyR inhibitor FLA365 or RyR siRNA both rescued these effects. Taken together, these preclinical studies suggest that mutations in *Ryr1* and *Fmr1*, two genes shown to be involved in neuronal Ca^2+^ handling, exert ASD-like behavioral and neuroarchitecture consequences in mice. Overall, these studies indicate that both genetic and environmental perturbation of neuronal Ca^2+^ homeostasis may contribute to aberrant synaptogenesis, dendritic arborization, and ultimately ASD-like behaviors.

### 2.2. Parvalbumin (PVALB)

Parvalbumin (PVALB) is a Ca^2+^-buffering protein primarily expressed in the γ-aminobutyric acid (GABA) positive interneurons of the brain that exhibit rapid burst-firing activity and are heavily dependent on intracellular Ca^2+^-handling (Ruden et al., 2021). While dysfunction of PVALB^+^ neurons is well-known to contribute to aberrant neurodevelopmental processes and ASD, the role of PVALB in maintaining the integrity of intracellular Ca^2+^ signaling pathways and its potential contribution to ASD has received less attention (Ruden et al., 2021). Interestingly, *Pvalb*^–/–^ mice are known to exhibit an ASD-like behavioral phenotype (Wöhr et al., 2015). Recently, Janickova et al. (2020) explored the role of PVALB in regulating neuron morphology and dendritic arborization by utilizing a *Pvalb*^–/–^ mouse strain in which EGFP expression was under the control of the *Pvalb* driver that allowed for visualization of PVALB*^+^* neurons even in the absence of functional *Pvalb* expression (Janickova and Schwaller, 2020; Janickova et al., 2020). Interestingly, loss of PVALB function resulted in increased cell soma and mitochondrial size primarily in regions rich in PVALB*^+^* neurons, such as the thalamic reticular nucleus (TRN), the molecular layer interneurons (MLI) of the cerebellum, the prefrontal cortex, and the striatum (Janickova and Schwaller, 2020; Janickova et al., 2020). Furthermore, loss of PVALB function was associated with dendritic hypertrophy in the dentate gyrus, the striatum, and the MLI, as well as by a shift of mitochondria from the central compartment of the cell to the subplasmalemmal region (Janickova and Schwaller, 2020). Taken together, these studies suggest that the impaired Ca^2+^ buffering brought about by the absence of PVALB may result in a compensatory proliferation and subplasmalemmal relocation of mitochondria to maintain the rapid Ca^2+^ dynamics these neurons rely on (Janickova and Schwaller, 2020; Ruden et al., 2021). Ultimately, this may result in enhanced dendritic arborization and oxidative stress. Although further studies are imperative, these data provide valuable insights into how PVALB-mediated Ca^2+^ dysfunction may induce ASD-relevant neurobiological correlates.

### 2.3. Inositol triphosphate receptors (IP_3_R)

G protein-coupled receptor (GPCR)-mediated IP_3_R Ca^+2^ signaling pathways comprise critical components of the intracellular Ca^+2^ handling machinery with potential implications in ASD (Berridge, 2009; Taylor and Tovey, 2010). For instance, the *IP3R2* has been shown to affected by *de novo* copy number variants in ASD patient cohorts, while recently *Ip3R2*^–/–^ mutant mice and astrocyte-specific *Ip3R2* conditional knockout mice display ASD-like behaviors (Gilman et al., 2011; Wang Q. et al., 2021). Interestingly, *ex vivo* studies in human fibroblasts derived from patients with rare, monogenic forms of ASD (i.e., fragile X syndrome; FXS and tuberous sclerosis; TS) showed that ATP-evoked GPCR-mediated Ca^2+^ release from the IP_3_Rs was diminished in ASD fibroblasts (Schmunk et al., 2015). In a follow-up study, Schmunk et al. (2017) extended their findings by using fibroblasts from patients with sporadic ASD, as well as two additional monogenic forms of ASD (i.e., Prader–Willi syndrome and Rett syndrome), and observed a similar impaired IP_3_R-mediated Ca^2+^ signaling. Taken together, these studies suggest that depressed Ca^2+^ release through IP_3_R signaling may disrupt neurodevelopment. To our knowledge these studies have not been replicated in neural cells or *in vivo* models, but provide mechanistic insights into the putative implication of IP_3_Rs in the neurobiology of ASD.

### 2.4. Neuronatin (NNAT) and other genes

Neuronatin (NNAT) is a developmentally regulated ER resident protein and negative regulator of SERCA that is expressed in the brain’s PVALB + GABAergic neurons; NNAT has also been implicated in abnormal neurodevelopment, including ASD and Angelman Syndrome (AS) (Pitale et al., 2017; Vatsa et al., 2019). The miR-708, an NNAT downregulator, has been involved in the atypical Ca^2+^ signaling processes observed in the maternal-ubiquitin protein ligase E3A (*Ube3a*) deficient mouse model for AS (Vatsa et al., 2019). UBE3A plays a role in the proteasome-mediated degradation of proteins in neurons, and has thus been implicated in ASD and AS (Glessner et al., 2009; Williams et al., 2010; Yi et al., 2015; Xu et al., 2018; Lopez et al., 2019). Recently, Vatsa et al. (2019) identified miR-708 to be significantly downregulated in the cortex of maternal-*Ube3a*-deficient AS mice and showed that miR-708 regulates intracellular Ca^2+^ homeostasis by targeting NNAT (Vatsa et al., 2019). Taken together, these findings suggest that NNAT/miR-708-mediated aberrations in intracellular Ca^2+^ signaling may be involved in ASD/AS pathogenesis.

Interestingly, targeted sequencing and integrative analysis of 3,195 Chinese probands with several neurodevelopmental disorders exposed novel candidate genes involved in ASD, including three with relevance to Ca^2+^ homeostasis, namely: *RYR3* [*discussed in* the Section “2.1. Ryanodine receptors (RyRs) and the fragile X messenger ribonucleoprotein 1 (FMR1)”], ubiquitin protein ligase E3 (*UBR3*), and filamin A (*FLNA*) (Wang T. et al., 2021). UBR3 inhibits the function of alpha 1C subunit of L-type voltage-dependent Ca^2+^ channel (Ca_*v*_1.2) *via* the ubiquitin-proteasome protein degradation pathway and has been identified as a modulator of Ca^2+^ -induced Ca^2+^ release (CIRC) (Ma et al., 2020). FLNA is an actin-binding protein which regulates cytoskeletal remodeling and is regulated by Ca^2+^ and calmodulin, and has been shown to interact with FMR1 in long term memory processes in *Drosophila* (Nakamura et al., 2005; Bolduc et al., 2010; Rosa et al., 2019). Overall, these findings further support a role for intracellular Ca^2+^ homeostasis in ASD pathogenesis, although further research is considered imperative to confirm the contribution of these genes in neurodevelopment.

### 2.5. Ca2^+^ signaling in astrocytes

It is well established that Ca^2+^ signaling is also prevalent in astrocytes; while astrocytic dysfunction has been implicated in the pathophysiology of ASD, the precise mechanisms by which astrocytes contribute to disease progression and symptomatology remain elusive (Blanco-Suárez et al., 2017). The onset of ASD pathology is typically concurrent with neurodevelopmental astrocyte proliferation (Berger et al., 2013; Sigaard et al., 2016). Allen et al. (2022) sought to investigate the putative role of astrocytes in ASD pathologyClick or tap here to enter text. Upon harvesting astrocytes from organoids created by induced pluripotent stem cells (iPSCs) from ASD patients (Allen et al., 2022). Proteomic analysis revealed that “Ca^2+^ binding” processes were highly enriched in the altered protein networks observed in these ASD astrocytes. Follow-up two-photon live-cell imaging confirmed an exaggerated ATP-induced Ca^2+^ response in these ASD astrocytes. To investigate putative behavioral effects of Ca^2+^ disruption in ASD astrocytes, human-derived ASD astrocytes were implanted into mice at P1-3, thus generating ASD astrocyte chimeric mice. Engrafted human ASD astrocytes were found to exhibit aberrant Ca^2+^ fluctuations, as well as to result in ASD-relevant behaviors (i.e., enhanced repetitive behaviors in the marble burying test and impaired fear learning). Given the exaggerated Ca^2+^ response observed in ASD astrocytes, it was predicted that inhibition of IP_3_Rs would possibly restore Ca^2+^ signaling and function. Intriguingly, IP_3_R-knockdown in ASD astrocytes rescued the exaggerated Ca^2+^ response, hippocampal neuron network firing, and deficits in fear memory observed in chimeric mice (Allen et al., 2022). Overall, these data provide deep insights into the contribution of astrocytic Ca^2+^ dysregulation in the pathophysiology of ASD.

## 3. Schizophrenia (SCZ)

Schizophrenia is a brain disorder characterized by a constellation of symptoms including hallucinations, negative affect, and cognitive deficits (McCutcheon et al., 2020). The Disrupted in Schizophrenia–1 (DISC-1) protein is involved in numerous neuronal processes, including the regulation of dendrite morphology and neuronal migration during development (Balu and Coyle, 2011). Recent studies have suggested that DISC-1 is involved in Ca^2+^ regulation *via* the mitochondria-associated membranes (MAMs) which comprise physical connections formed between the ER IP_3_Rs and mitochondrial voltage-dependent anion channels (VDAC) that are involved in the transfer of Ca^2+^ and molecular stress signals between these two organelles (Park et al., 2017, 2015; van Vliet and Agostinis, 2018; Barazzuol et al., 2021; Means and Katz, 2021). Recent findings suggest that DISC-1 localizes to the MAM in mouse neurons, and specifically binds IP_3_R1 to reduce ligand-binding and subsequent Ca^2+^ transfer to the mitochondria in primary cortical neurons (Park et al., 2017). Upon DISC-1 dysfunction, IP_3_R1-mediated Ca^2+^ release into the MAM is disinhibited, causing a buildup of mitochondrial Ca^2+^ that leads to oxidative stress that ultimately impairs mitochondrial function (Park et al., 2017). Interestingly, neuronal oxidative stress has been implicated in the pathogenesis of SCZ (Emiliani et al., 2014). Taken together, this experimental evidence suggests that DISC-1 is involved in the dysregulation of Ca^2+^ handling in the MAMs, causing downstream mitochondrial Ca^2+^ hyper-accumulation and oxidative stress, shining a light on a novel mechanism by which DISC-1 may contribute to SCZ pathogenesis.

Darier’s disease is a skin condition characterized by persistent wart-like skin patches, which is due to a mutation in the *SERCA2* gene that subsequently leads to Ca^2+^ dysfunction (Cooper and Burge, 2003). Interestingly, Darier’s disease patients have a significantly increased risk for SCZ, providing a causative link between *SERCA2* and neurodevelopmental processes (Tang et al., 2010). Recently, Nakajima et al. (2021) generated a brain-specific heterozygous *Serca2* loss-of-function mouse model (i.e., hetero cKO) to investigate how developmental hypofunction of *Serca2* may affect SCZ-relevant behavioral and neurobiological processes (Nakajima et al., 2021). As expected, both primary hippocampal neurons and ER membranes isolated from the brain of hetero cKO mice exhibited impaired Ca^2+^ uptake (Nakajima et al., 2021). Hetero cKO mice exhibited impaired fear memory and enhanced exploratory behavior; moreover, microdialysis studies suggested that *Serca2* hypofunction induces a hyperdopaminergic state in the nucleus accumbens (NAC) (Nakajima et al., 2021), echoing the neurochemical dopaminergic hallmarks of SCZ (McCutcheon et al., 2020). Taken together, these findings support the notion that developmental hypofunction of the *Serca2* and subsequent aberrant intracellular Ca^2+^ handling induces SCZ-relevant behavioral and neurochemical effects.

Interestingly, recent evidence suggests an association between RyRs and SCZ. An exome sequencing study of childhood-onset SCZ patients, identified *de novo* variants of *RYR2*, which the authors highlight as a strong candidate gene given the role of RyRs in neurodevelopmental processes (Ambalavanan et al., 2016), further underscoring the putative role of RyRs in the neurobiology of neurodevelopmental disorders.

## 4. Attention-deficit hyperactivity disorder (ADHD)

Attention-deficit hyperactivity disorder is a neurodevelopmental disorder that is characterized by impaired attention, locomotor hyperactivity, and impulsive behaviors (Sharma and Couture, 2014). Preclinical evidence suggests that ADHD is associated with impaired intracellular Ca^2+^ handling; for instance, the spontaneously hypertensive rat (SHR) model of ADHD has been shown to exhibit impaired brain plasma membrane Ca^2+^ uptake (Horn et al., 1995; Lehohla et al., 2001). Further preclinical evidence has shown that knockout of the G-protein subunit Gβ5 (encoded by the gene *Gnb5*) elicits a pronounced ADHD-like hyperactive phenotype in mice (Xie et al., 2012). Moreover, a *GNB5* mutation (i.e., GNB5 S81L) associated with impaired termination of DA2 receptor signaling was reported in a Saudi family presenting speech impairments and a variable ADHD diagnosis, providing initial clinical evidence for the putative role of GNB5 in the neurobiology of ADHD (Shamseldin et al., 2016). Interestingly, a recent study highlighted the role of GNB5 in store-operated Ca^2+^ entry (SOCE) (Kang et al., 2018). Upon depletion of ER Ca^2+^ stores, stromal interaction molecule 1 (STIM1), an ER Ca^2+^ sensor, forms a complex with the plasma membrane calcium release-activated calcium channel protein 1 (ORAI1) to initiate extracellular Ca^2+^ entry (Srikanth and Gwack, 2012). Kang et al. (2018) found that GNB5 expression enhances SOCE *in vitro*. Notably, the ability of GNB5 to enhance SOCE was found to depend on STIM1 function suggesting that GNB5 may interact with the ER Ca^2+^-sensing machinery to regulate Ca^2+^ homeostasis, although further studies are needed to determine the precise mechanisms that may underlie this process.

## 5. Conclusion

In the context of this mini-review, we have highlighted recent advances supporting the implication of prominent ER and cytosolic Ca^2+^ regulators (i.e., SERCA2, IP_3_Rs, RyRs, PVALB, NNAT) in the neurobiology of brain disorders with a strong neurodevelopmental component (Figure 1 and Table 1). Disease progression of monogenic brain disorders (e.g., AS, FXS) may be dependent on specific gene interactions with intracellular Ca^2+^ signaling mechanisms, whereas sporadic cases of SCZ, ASD, and ADHD may arise from polygenic variations that ultimately converge to the disruption of intracellular Ca^2+^ homeostasis and concomitant impairment of neuronal function. Further preclinical and clinical investigation is considered imperative to confirm and/or expand upon these intriguing discoveries in order to gain deep insights into the cellular and molecular Ca^2+^-dependent neurodevelopmental processes that are compromised in these debilitating brain diseases.

**TABLE 1:** Studies focusing on notable genes implicated in the pathophysiology of neurodevelopmental disorders and summarized findings on intracellular Ca^2+^ signaling, neuronal function, and behavior.

Gene	Study	Gene manipulation	Disorder indication	Effect on Ca^2+^	Effect on neuronal function	Effect on behavior
*Ryr1*	Keil et al., 2019	Humanized GoF *Ryr1* T4826I mutation	ASD	N/A	↑ Dendritic complexity	↓ Sociability
*Fmr1*	Keil et al., 2019	CGG-repeat GoF *Fmr1*	ASD	N/A	↑ Dendritic complexity	N/A
*Pvalb*	Janickova et al., 2020	*Pvalb* KO mouse	ASD	N/A	↑ Soma and dendrite size	N/A
*Nnat*	Vatsa et al., 2019	miR-207	ASD/AS	↓ Intracellular Ca^2^^+^*CPSTABLEENTER*↓ CaMKIIα phosphorylation	N/A	Maternal-*Ube3a* deficient mouse model for AS
DISC-1	Park et al., 2015, 2017	DISC-1 KD	SCZ	↓ MAM Ca^2+^ transfer	N/A	N/A
*Serca2*	Nakajima et al., 2021	Brain-specific heterozygous knockout		↓ER Ca^2+^ uptake	↑ NAc DA	↑ Exploratory behavior ↓ Fear memory
*Gnb5*	Xie et al., 2012	GNB5 KO mouse	ADHD	N/A	N/A	↑ Hyperactivity
*GNB5*	Kang et al., 2018	GNB5 overexpression in HEK293T cells	ADHD	↑ SOCE	N/A	N/A

GoF, gain of function; KD, knock-down; KO, knock-out.

## Author contributions

BK conducted the primary literature search and wrote first draft of the manuscript. KK, JT, CM, and HO wrote sections of the manuscript. PP formulated the concept and supervised the writing of the manuscript. All authors contributed to manuscript editing, revision, read, and approved the submitted version.
